# Psychometric evaluation of the Polish adaptation of the Hill-Bone Compliance to High Blood Pressure Therapy Scale

**DOI:** 10.1186/s12872-016-0270-y

**Published:** 2016-05-10

**Authors:** Izabella Uchmanowicz, Beata Jankowska-Polańska, Anna Chudiak, Anna Szymańska-Chabowska, Grzegorz Mazur

**Affiliations:** Department of Clinical Nursing, Faculty of Health Science, Wroclaw Medical University, 5 Bartla Street, 51-618 Wroclaw, Poland; Department of Internal Medicine, Occupational Diseases and Hypertension, Wroclaw Medical University, 213 Borowska Street, 50-556 Wroclaw, Poland

**Keywords:** Hypertension, Adherence, Hill-Bone Compliance to High Blood Pressure Therapy Scale, Internal consistency, Reliability

## Abstract

**Background:**

Development of simple instruments for the determination of the level of adherence in patients with high blood pressure is the subject of ongoing research. One such instrument, gaining growing popularity worldwide, is the Hill-Bone Compliance to High Blood Pressure Therapy.

The aim of this study was to adapt and to test the reliability of the Polish version of Hill-Bone Compliance to High Blood Pressure Therapy Scale.

**Methods:**

A standard guideline was used for the translation and cultural adaptation of the English version of the Hill-Bone Compliance to High Blood Pressure Therapy Scale into Polish. The study included 117 Polish patients with hypertension aged between 27 and 90 years, among them 53 men and 64 women. Cronbach’s alpha was used for analysing the internal consistency of the scale.

**Results:**

The mean score in the reduced sodium intake subscale was M = 5.7 points (standard deviation SD = 1.6 points). The mean score in the appointment-keeping subscale was M = 3.4 points (standard deviation SD = 1.4 points). The mean score in the medication-taking subscale was M = 11.6 points (standard deviation SD = 3.3 points). In the principal component analysis, the three-factor system (1 – medication-taking, 2 – appointment-keeping, 3 – reduced sodium intake) accounted for 53 % of total variance. All questions had factor loadings > 0.4. The medication-taking subscale: most questions (6 out of 9) had the highest loadings with Factor 1. The appointment-keeping subscale: all questions (2 out of 2) had the highest loadings with Factor 2. The reduced sodium intake subscale: most questions (2 out of 3) had the highest loadings with Factor 3. Goodness of fit was tested at chi^2^ = 248.87; *p* < 0.001. The Cronbach’s alpha score for the entire questionnaire was 0.851.

**Conclusion:**

The Hill-Bone Compliance to High Blood Pressure Therapy Scale proved to be suitable for use in the Polish population. Use of this screening tool for the assessment of adherence to BP treatment is recommended.

## Background

Hypertension, or high blood pressure (HBP), is considered the most common disorder in the general population. The World Health Organization (WHO) Report for 2008 states that 40 % of the world’s population over the age of 25 have high blood pressure [1]. Worldwide, HBP affects over 1.5 billion people, in Poland – approximately 8.6 million, i.e. 29 % of adults. According to estimates, in 2025, over 2 billion people will suffer from HBP [2]. Despite the wealth of knowledge and the advances in pharmaceutical treatment and hypotensive drugs, the effectiveness of HBP treatment in Poland is as low as 5–15 % [3]. Non-adherence to medications is considered one of the largest related issues [4, 5]. Several methods can be used to test compliance and adherence, including pharmacological, clinical and physical ones. The first method consists of determining the concentrations of drugs or their metabolites in serum or in urine. The clinical methods are based on monitoring follow-up appointment-keeping. It is assumed that patients who are cooperative and follow their physicians’ advice tend to keep their follow-up appointments. The remaining methods used for determining adherence and compliance are physical ones, consisting in counting the tablets taken or using tablet-counting systems. These methods are not, however, reliable, as the presentation of an empty medicine package to the physician is no proof that all the doses were taken as prescribed. Patients may deliberately dispose of the medicine, which artificially increases the score [6–8]. Remote observation is an important aspect of compliance and adherence assessment among patients with HBP. After starting pharmaceutical treatment, follow-up visits should take place every 2–4 weeks to enable assessment of the drugs’ effectiveness and any side effects. Once blood pressure falls to the desired values, these follow-ups may be planned at longer intervals, every few months [9–11]. Every 2 years, all patients should be checked for risk factors and end-organ complications of HBP [12]. Physicians‘lack of knowledge and patients‘lack of awareness account for about 70 % of non-adherence, indicating the necessity to improve physician education and patient involvement [13, 14]. Therefore, the researchers work on simple instruments to assess compliance and adherence to treatment in this group of patients. One such instrument, increasing in popularity worldwide, is the Hill-Bone Compliance to High Blood Pressure Therapy Scale [15]. In its current version, the Hill-Bone Compliance to High Blood Pressure Therapy Scale comprises 14 questions that are grouped in the following sub-scales: reduced sodium intake (three items), appointment-keeping (two items), and medication-taking (nine items) [15]. The instrument has successfully passed validation and psychometric evaluation in many populations and cultures, and has undergone multiple translations and adaptations [16–19]. We decided to perform a cultural Polish adaptation of the Hill-Bone Compliance to High Blood Pressure Therapy Scale questionnaire and to validate it in a group of HBP patients. This paper presents the results of our validation study.

## Methods

### Questionnaire

#### The Polish adaptation of the Hill-Bone Compliance to High Blood Pressure Therapy Scale (HBCS)

The adaptation was performed using the standard methodology [20]. The Polish adaptation is based on the English-language version of the scale (Table 1) [15]. Having received the authors’ approval, the questionnaire was translated into Polish by two independent translators. Then, the translations were evaluated by a panel of experts, which comprised a cardiologist, a general practitioner, two specialist cardiology nurses and a psychologist. The panel verified the phrasing and meaning of all questions, as well as the clarity and correctness of the instructions. The version selected by the panel subsequently underwent back-translation and the result was submitted for approval by the authors of the original English version. Once approved, the preliminary version was used in a pilot study on a group of 30 patients diagnosed with HBP. The pilot study resulted in the final Polish version of the Hill-Bone Compliance to High Blood Pressure Therapy Scale, validated in the present study.

**Table 1:** The Hill-Bone Compliance to HBP Therapy Scale – the original English version

No.	Item	Response:
		1. All of the time 2. Most of the time 3. Some of the time 4. Never
1	How often do you forget to take your HBP medicine?	
2	How often do you decide NOT to take your HBP medicine?	
3	How often do you eat salty food?	
4	How often do you add salt to your food before you eat it?	
5	How often do you eat fast food?	
6	How often do you make the next appointment before you leave the doctor’s office?*	
7	How often do you miss scheduled appointments?	
8	How often do you forget to get prescriptions filled?	
9	How often do you run out of HBP pills?	
10	How often do you skip your HBP medicine before you go to the doctor?	
11	How often do you miss taking your HBP pills when you feel better?	
12	How often do you miss taking your HBP pills when you feel sick?	
13	How often do you take someone else’s HBP pills?	
14	How often do you miss taking your HBP pills when you are careless?	

#### Patients

The study was carried out at a private medical center in Wrocław, between March and September, 2015, and included 117 sequential patients diagnosed with HBP in accordance with the ESC criteria. The patients filled in the questionnaire during a regular follow-up visit. The refusal rate was 0 %, and the acceptance rate was 100 %. The only exclusion criterion was cognitive impairment and/or communication barriers interfering with questionnaire completion. The sample size was based on literature data, indicating that the minimum number of participants in a validation study should be five times the number of variables analyzed [21]. Therefore, a validation study of the Hill-Bone Compliance to High Blood Pressure Therapy Scale should include a minimum of 70 patients (5 × 14 statements). It was the representative sample of hypertensive patients in Poland, so it might be generalized to other patients. All patients gave informed written consent to participate in writing, and the study protocol was approved by the Independent Bioethics Committee of the Wrocław Medical University (decision no. KB 136/2015).

#### Statistical analysis

The results obtained were analyzed using Statistica 10 software (StatSoft, Tulsa, USA). The normality of variable distribution was verified using the Kolmogorov–Smirnov test, and the statistical characteristics of variables were presented using arithmetical means ± standard deviations (SD), median values and interquartile ranges (IQR). The statistical characteristics of discrete and qualitative variables were presented using number distributions (n). The internal consistency and the intraclass correlation coefficients of the Polish adaptation were assessed based on Cronbach’s alpha.

## Results

The socio-demographic and clinical characteristics of the 117 patients studied are summarized in Table 2. The sample included 53 men (45.30 %) and 64 women (54.70 %). The mean age was 60.7 years (SD 12.41). Most patients were married or living with a partner (66.67 %) and high-school educated (51.28 %). The mean time from diagnosis was 9.18 years (SD 7.4). The number of tablets taken daily was 2.61 (SD 3.26). The detailed characteristics of the patients are shown in Table 2. The Table 3 shows descriptive statistics from the Hill-Bone Compliance to High Blood Pressure Therapy Scale in the studied group.

The mean sodium intake score among the patients studied was M = 5.7 points (standard deviation SD = 1.6 points). The mean appointment-keeping score in the group was M = 3.4 points (standard deviation SD = 1.4 points). The mean medicine-taking score was M = 11.6 points (standard deviation SD = 3.3 points).

**Table 2:** Patients' socio-demographic and clinical data

Socio-demographic and clinical data	n	% of total
Sex		
Female	64	54.70
Male	53	45.30
Age		
M ± SD	60.77 ± 12.41	
Min ÷ Max	27 ÷ 90	
Marital status		
Married/living with a partner	78	66.67
Single	39	33.33
Education		
None or primary	32	27.35
High school	60	51.28
Higher vocational/College/University	25	21.37
Blood pressure value	Systolic	Diastolic
M ± SD	144.14 ± 13.69	87.85 ± 11.36
Min ÷ Max	120 ÷190	60 ÷ 120
Time from HBP diagnosis		
M ± SD	9.18 ± 7.4	
Min ÷ Max	1 ÷10	
JNC blood pressure classification		
Stage 1 hypertension	63	53.85
Stage 2 hypertension	36	30.77
Prehypertension	18	15.38

*n* number, *M ± SD* mean ± standard deviation, *Min ÷ Max* minimum ÷ maximum, *HBP* high blood pressure, *JNC* Joint National Committee

### The scale’s construct validity

The principal components of the HBSC scale were analyzed. The three-factor system (variables: 1 – medication-taking, 2 – appointment-keeping, 3 – reduced sodium intake) accounts for 53 % of total variance. All questions have factor loadings >0.4. The medication-taking subscale: most questions (6 out of 9) have the highest loadings with Factor 1. The appointment-keeping subscale: all questions (2 out of 2) have the highest loadings with Factor 2. The reduced sodium intake subscale: most questions (2 out of 3) have the highest loadings with Factor 3. Goodness of fit was tested at chi^2^ = 248.87; *p* < 0.001 (Table 4). The instrument shows appropriate construct validity and no questions need to be excluded.

**Table 3:** Descriptive statistics from the Hill-Bone Compliance to High Blood Pressure Therapy Scale in the studied group

Question		M ± SD	All the time [%]	Most of the time [%]	Sometimes [%]	Never [%]
3	How often do you eat salty food?	2.41 ± 0.76	6.84	53.85	29.06	9.40
4	How often do you add salt to your food before you eat it?	1.93 ± 0.85	35.04	41.03	19.66	4.27
5	How often do you eat fast food?	1.40 ± 0.61	63.25	29.06	3.42	0.85
Reduced sodium intake		5.68 ± 1.60				
6	How often do you make the next appointment before you leave the doctor’s office?	2.75 ± 1.05	11.97	29.06	22.22	29.91
7	How often do you miss scheduled appointments?	1.30 ± 0.61	74.36	19.66	2.56	1.71
Appointment-keeping		3.41 ± 1.45				
1	How often do you forget to take your HBP medicine?	1.39 ± 0.64	67.52	26.50	3.42	1.71
2	Jak często decyduje się Pan/Pani nie brać leków na nadciśnienie? [How often do you decide NOT to take your HBP medicine?]	1.35 ± 0.62	70.94	21.37	5.13	0.85
8	How often do you forget to get prescriptions filled?	1.30 ± 0.50	70.94	25.64	1.71	0.00
9	How often do you run out of HBP pills?	1.42 ± 0.56	60.68	34.19	3.42	0.00
10	How often do you skip your HBP medicine before you go to the doctor?	1.25 ± 0.43	72.65	23.93	0.00	0.00
1	How often do you miss taking your HBP pills when you feel better?	1.39 ± 0.68	68.38	26.50	0.85	3.42
12	How often do you miss taking your HBP pills when you feel sick?	1.33 ± 0.60	71.79	21.37	4.27	0.85
13	How often do you take someone else’s HBP pills?	1.17 ± 0.53	86.32	7.69	1.71	1.71
14	How often do you miss taking your HBP pills when you are careless?	1.25 ± 0.53	77.78	16.24	4.27	0.00
Medicine-taking		11.62 ± 3.32				

*HBP* high blood pressure, *M ± SD* mean ± standard deviation

### The scale’s criterion validity

Analysis of correlation with external criteria (systolic and diastolic blood pressure) – Spearman’s rank correlation coefficients (Table 5). The instrument has unique criterion validity, which is a complement to the blood pressure measurement and not its substitute.

### Scale reliability

Internal consistency was tested using Cronbach’s alpha, and the intraclass correlation coefficient was determined. The Cronbach’s alpha score for the whole questionnaire was 0.80, and for the medicine-taking subscale it was 0.78. Cronbach’s alpha was not calculated for the remaining subscales due to the insufficient number of questions. Coefficients of correlation with the total score ranged from 0.14 (question 3) to 0.653 (question 10). The intraclass correlation coefficient for the whole questionnaire was 0.851 (Table 6).

**Table 4:** Factor loadings for each question in the questionnaire

Question	Factor 1	Factor 2	Factor 3
Question_2	**0.35**	−0.04	0.20
Question_5	**0.51**	0.22	0.41
Question_8	**0.59**	0.29	0.07
Question_9	**0.83**	0.08	0.05
Question_11	**0.88**	0.16	−0.04
Question_12	**0.67**	0.18	0.09
Question_13	**0.47**	0.01	0.09
Question_1	0.11	**0.80**	0.02
Question_6	−0.22	**0.44**	0.38
Question_7	0.54	**0.65**	−0.07
Question_10	0.31	**0.72**	0.30
Question_14	0.05	**0.76**	−0.09
Question_3	0.11	−0.15	**0.71**
Question_4	−0.05	0.17	**0.74**
Expl.Var	3.33	2.61	1.55
Prp.Totl	0.24	0.19	0.11

**1**-How often do you forget to take your HBP medicine? **2-**How often do you decide NOT to take your HBP medicine? **3-**How often do you eat salty food? **4-**How often do you add salt to your food before you eat it? **5-**How often do you eat fast food? **6-**How often do you make the next appointment before you leave the doctor’s office? **7-**How often do you miss scheduled appointments? **8-**How often do you forget to get prescriptions filled? **9-**How often do you run out of HBP pills? **10-**How often do you skip your HBP medicine before you go to the doctor? **11-**How often do you miss taking your HBP pills when you feel better? **12-**How often do you miss taking your HBP pills when you feel sick? **13-**How often do you take someone else’s HBP pills? **14-**How often do you miss taking your HBP pills when you are careless?

## Discussion

The aim of the study was to prepare a Polish adaptation of the Hill-Bone Compliance to High Blood Pressure Therapy Scale and to validate the instrument in a group of HBP patients. The Hill-Bone Compliance to High Blood Pressure Therapy Scale showed sufficient psychometric quality in different aspects of acceptability, reliability and validity. The validation was based on determining a routine psychometric characteristic – Cronbach’s alpha, measuring the instrument’s internal consistency. The internal consistency of the Polish adaptation of the Hill-Bone Compliance to High Blood Pressure Therapy Scale was tested at 0.851. This value is similar to the original instrument’s internal consistency and to values obtained in validation studies of other language versions and cultural adaptations performed in Turkey (0.72) [15, 16], South Africa (0.77) [17] and Germany (0.73) [18]. However, in the adaptation by Krauser-Wood et al. it was found that the scale has sufficient internal consistency and factorial construct validity only for the medication-taking subscale [19]. Therefore, the authors recommend studying adherence using only this subscale (Hill-Bone Scale Short Form – SF). In the Polish adaptation, the Hill-Bone Scale was shown to have sufficient construct and criterion validity, which means HBSC scores were predictive of BP outcomes and were significantly correlated with other theoretically selected variables. The HBP scale also demonstrated high internal consistency when used in South Africa or when translated into Turkish [16, 17]. As to the interpretation of the results obtained, it should be emphasized that the psychometric qualities of the Polish adaptation of the Hill-Bone Compliance to High Blood Pressure Therapy Scale are superior to those obtained in other language versions and cultural adaptations. For example, the German validation of the HBP scale showed floor effects and poor ability to predict medication adherence for nearly every third participant [18]. According to some authors, the optimum internal consistency is indicated by Cronbach’s alpha at ≥0.90; values ≥0.80 are considered good, ≥ 0.70 – acceptable, ≥ 0.60 – questionable, ≥ 0.50 – poor and <0.50 – unacceptable [22]. Therefore, the Polish adaptation of the Hill-Bone Compliance to High Blood Pressure Therapy Scale questionnaire can be considered an instrument with good psychometric qualities. A reliable and valid adherence measurement based on the patient’s self-report may be helpful in daily practice.

**Table 5:** Spearman's rank correlation coefficients for the scale total and the medication taking subscale score on the one hand, and systolic and diastolic blood pressure on the other

Variable	Systolic blood pressure		Diastolic blood pressure	
	R	*p*-value	R	*p*-value
Medicine taking	−0.039	0.679	−0.015	0.873
Total score	0.017	0.859	0.049	0.599

R – Spearman's rank correlation coefficient; p-value – statistical significance coefficient

**Table 6:** Internal consistency and intraclass correlation coefficients for each question

Question	Correlation with the total score	Cronbach's alpha with the question excluded	Intraclass correlation coefficient
Question 1	0.471	0.768	0.842
Question 2	0.209	0.788	0.852
Question 3	0.140	0.791	0.820
Question 4	0.230	0.784	0.886
Question 5	0.541	0.758	0.830
Question 6	0.157	0.808	0.826
Question 7	0.599	0.756	0.787
Question 8	0.505	0.765	0.807
Question 9	0.543	0.761	0.855
Question 10	0.653	0.746	0.840
Question 11	0.582	0.760	0.888
Question 12	0.504	0.763	0.861
Question 13	0.320	0.780	0.807
Question 14	0.380	0.774	0.806

**1-**How often do you forget to take your HBP medicine? **2-**How often do you decide NOT to take your HBP medicine? **3-**How often do you eat salty food? **4-**How often do you add salt to your food before you eat it? **5-**How often do you eat fast food? **6-**How often do you make the next appointment before you leave the doctor’s office? **7-**How often do you miss scheduled appointments? **8-**How often do you forget to get prescriptions filled? **9-**How often do you run out of HBP pills? **10-**How often do you skip your HBP medicine before you go to the doctor? **11-**How often do you miss taking your HBP pills when you feel better? **12-**How often do you miss taking your HBP pills when you feel sick? **13-**How often do you take someone else’s HBP pills? **14-**How often do you miss taking your HBP pills when you are careless?

## Conclusions

The Hill-Bone Compliance to High Blood Pressure Therapy Scale proved to be suitable for use in the Polish population. The use of this screening tool for the assessment of adherence to BP treatment is recommended.

## Ethics approval and consent to participate

The study protocol was approved by the Independent Bioethics Committee of the Wroclaw Medical University (decision no. KB 136/2015). All participants gave written informed consent after thorough explanation of the procedures involved.

## Consent for publication

Not applicable

## Availability of data and materials

The datasets supporting the conclusions of this article are included within the article.

## Abbreviations

BP: blood pressure
HBP: high blood pressure
ESC: European Society of Cardiology
SD: standard deviation
IQR: interquartile ranges
WHO: World Health Organization
HBSC scale: Hill-Bone Compliance to High Blood Pressure Therapy Scale
SF: Hill-Bone scale short form
